# DDIT4 overexpression associates with poor prognosis in lung adenocarcinoma

**DOI:** 10.7150/jca.60118

**Published:** 2021-09-03

**Authors:** Li Song, Zhiyao Chen, Menghua Zhang, Mingli Zhang, Xiaoyun Lu, Chunsun Li, Liyan Miao

**Affiliations:** 1 Department of Pharmacy, The First Affiliated Hospital of Soochow University, Suzhou, Jiangsu, China, 215006.; 2 Department of Oncology, Affiliated Tumor Hospital of Nantong University, Nantong 226361, Jiangsu, China.; 3 Department of Pathology, Affiliated Tumor Hospital of Nantong University, Nantong 226361, Jiangsu, China.

**Keywords:** DDIT4, Lung adenocarcinoma, Prognosis

## Abstract

**Objectives:** DNA damage inducible transcript 4 (DDIT4) plays a key role in different cancers, but the role of DDIT4 in lung adenocarcinoma (LUAD) is not completely understood. The aim of this study was to evaluate the utility of DDIT4 as a prognostic biomarker for LUAD.

**Methods:** First, DDIT4 mRNA expression in LUAD cell lines (A549, H1299 and HBE) and tissues (89 cases) was assessed by RT-PCR. Next, DDIT4 protein expression in LUAD tissues and normal tissues was assessed by immunohistochemistry (75 cases). Then, the correlation between DDIT4 expression and overall survival was analyzed using the Kaplan-Meier method. After that, we verified the utility of the DDIT4 gene as a prognostic marker of lung cancer in the TCGA database (1133 cases). Finally, the possible mechanism of the DDIT4 gene as a prognostic marker of LUAD was preliminarily explored.

**Results:** mRNA levels of DDIT4 in HBE cells were significantly lower than in A549 and H1299 cells (P<0.05), and expression of the DDIT4 gene in cancer tissues was significantly higher than in adjacent tissues (P<0.0001). Immunohistochemical staining results showed that high expression of DDIT4 accounted for approximately 68.0% of LUAD tissues. DDIT4 expression was significantly correlated with differentiation (P < 0.05). However, it was not correlated with sex, age, smoking, tumor size, lymph node metastasis, or TNM stage (P>0.05). The survival analysis demonstrated that high DDIT4 expression was correlated with shorter overall survival (P < 0.05). Univariate and multivariate analyses indicated that DDIT4 was an independent predictor of overall survival for LUAD, which was confirmed by data from the TCGA database. Finally, we found that DDIT4 gene expression was significantly increased in the hypoxic environment compared to the normal oxygen environment, indicating that the DDIT4 gene may play an important role in the hypoxic microenvironment of tumor tissue.

**Conclusion:** High expression levels of DDIT4 correlated with poor overall survival in patients with LUAD, and DDIT4 was an independent predictor of overall survival. These findings provide new insight for understanding the development of LUAD.

## Introduction

Lung cancer is the most common cancer and the leading of cancer-related mortality worldwide 1. Non-small cell lung cancer (NSCLC), including lung adenocarcinoma (LUAD), lung squamous carcinoma (LUSC), and large cell carcinoma (LCC), accounts for approximately 80% of all lung cancer cases 2. Although targeted therapy has provided some disease control in LUAD patients over the past decades, overall survival is far from satisfactory 3. Cancer metastasis and recurrence continue to be major challenges for clinicians. Therefore, there is a need to identify novel molecular biomarkers to provide clinicians with a better assessment of recurrence risk and prognosis.

DDIT4 (DNA damage-inducible transcript 4), also known as REDD1 (regulated in development and DNA damage responses 1), or RTP801, was discovered and cloned in 2002 by Shoshani and Ellisen, two independent research groups 4-5. DDIT4 expression is induced by hypoxia 6, heat shock and energy depletion 7. Dysfunction of DDIT4 has been associated with dozens of diseases, such as neurodegenerative disorders 8, preeclampsia 9, and diabetes 10.

Several works have shown that DDIT4 may play a key role in cancer as an oncogene. Chang et al. 11 found that DDIT4 upregulation associates with tumor progression and is an unfavorable prognostic factor in ovarian carcinoma. DDIT4 promotes proliferation and tumorigenesis through the p53 and MAPK pathways in gastric cancer 12. Some studies have indicated that overexpression of DDIT4 is also an adverse factor in acute myelocytic leukemia 13-14. However, the role of DDIT4 in LUAD is not completely understood.

To better understand the function of DDIT4 in LUAD, we focused on determining the levels of DDIT4 in different LUAD cell lines, tissues, and paraffin sections. Meanwhile, the association between DDIT4 levels and disease progression was investigated from immunohistochemical results and the TCGA database. Based on these results, we preliminarily explored the possible utility of the DDIT4 gene as a prognostic marker for LUAD.

## Materials and methods

### Reagents and materials

RNAiso Plus, PrimeScript RT reagent Kit with gDNA Eraser (Perfect Real Time) and SYBR Premix Ex Taq II (Tli RNaseH Plus) were purchased from TaKaRa Biotechnology Co., Ltd. (Dalian, China). PCR primers and probes were designed using Primer 5.0 and synthesized by Sangon Biotechnology Co. Ltd. (Shanghai, China). Human lung adenocarcinoma cell lines (A549 and H1299) and a human bronchial epithelial cell line (HBE) were purchased from the Chinese Academy of Sciences (Shanghai, China). Antibodies against DDIT4 were purchased from Atlas Antibodies AB (Sweden).

### Patients and tissue specimens

Tissues were collected from 89 primary LUAD patients treated with surgical resection from The First Affiliated Hospital of Soochow University between January 2016 and December 2018. All of the patients were pathologically confirmed to be LUAD, and all patients had not received prior treatment with chemotherapy or immunotherapy before surgical resection. In addition, patients with performance status (PS) of 0-2 were included, and patients with a history of tumor within 5 years and synchronous other malignant tumors were excluded. Follow-up visit data was updated through April 2021 by performing telephone and reviewing medical records. TNM staging was assessed according to the American Joint Committee on Cancer guidelines. Paraffin-embedded tissues of 75 LUAD patients were collected at the Affiliated Tumor Hospital of Nantong University between January 1, 2008, and December 31, 2013. Inclusion and exclusion criteria of these patients were consistent with the patients mentioned above. Follow-up visit data was updated through December 2018 by performing telephone and reviewing medical records. The clinical materials used for research purposes have been approved by the Institutional Research Ethics Committee.

### Cell culture

Human lung adenocarcinoma cell lines (A549 and H1299) and a human bronchial epithelial cell line (HBE) were purchased from the Chinese Academy of Sciences (Shanghai, China). HBE and H1299 cells were cultured in RPMI 1640 medium, and A549 cells were cultured in MEM (Gibco, Carlsbad, CA, USA). All media were supplemented with 10% fetal bovine serum (FBS) (HyClone, Thermo Fisher Scientific, Waltham, MA, USA) and 100 U/mL penicillin-streptomycin mixture (GibCo BRL, Grand Island, NY, USA). All cell lines were grown in a humidified 5% CO_2_ incubator at 37 °C. For hypoxic exposure, tumor cells were incubated in a hypoxic incubator with 1% O_2_, 5% CO_2_ and 94% N_2_.

### Reverse transcription-PCR (RT-PCR)

First, total RNA was extracted from tissues and cells using RNAiso Plus reagent, and then the extracted mRNA was reverse transcribed into cDNA using a PrimeScript RT reagent Kit with gDNA Eraser (Perfect Real Time). Thereafter, real-time PCR was applied using SYBR® Premix Ex Taq™ II (Tli RNaseH Plus) and a 7500 Real Time PCR System (Applied Biosystems, Carlsbad, CA, USA). Expression of target genes was determined relative to* β*-actin, and analysis was calculated by the 2^-ΔΔCt^ method. The following primer sequences were used:

- DDIT4, F: 5′-AGGGGTTTGACCGCTCCA-3′ and R: 5′- CCAGGTAAGCCGTGTCTTCC - 3′;
- β-actin, F: 5′- GCACAGAGCCTCGCCTTT -3′ and R: 5′-CCCACCATCACGCCCTG - 3′.

### Immunohistochemistry

Paraffin-embedded sections were routinely dewaxed and hydrated with gradient ethanol. Sections were dewaxed, dehydrated and rehydrated. Then, sections were probed overnight with primary antibody against DDIT4 (1:200 dilution, Atlas Antibodies AB, Sweden) and with the secondary antibody at ambient temperature for 30 min. Finally, all sections were rinsed with water, counterstained with hematoxylin, dehydrated and coverslipped. Staining intensity was scored from 0-3 (0 = negative; 1 = weak; 2 = medium; 3 = strong). The percentage of positively stained cells was scored from 1-4: 0 (0%), 1 (1-25%), 2 (26-50%), 3 (51-75%) and 4 (76-100%). Multiplication of the percentage of positive cells and the intensity resulted in a score. Next, we split the cases into two groups, those with low expression (with scores ≤6) and those with high expression (with scores>6).

### TCGA database and data collection

Expression of the DDIT4 gene in the TCGA lung cancer database was extracted. We used Graph Pad software (Graph Pad Software, Inc., La Jolla, CA, USA) to draw the scatter plots. The survival curve of the DDIT4 gene was generated with Kaplan-Meier Plotter.

### Statistical analysis

Data were analyzed in SPSS 19.0 software (IBM Corporation, Armonk, NY, USA). The Chi-squared test and Fisher's exact test were used to analyze counting data, and Student's t-test or Mann-Whitney U test was used to analyze measurement data if appropriate. Data were analyzed using the two-tailed t-test. Kaplan-Meier (K-M) and log-rank analyses were used to analyze survival curves. A P-value <0.05 was considered statistically significant in all analyses.

## Results

### Expression of DDIT4 is upregulated in lung adenocarcinoma cell lines and tissues

**Figure 1:**
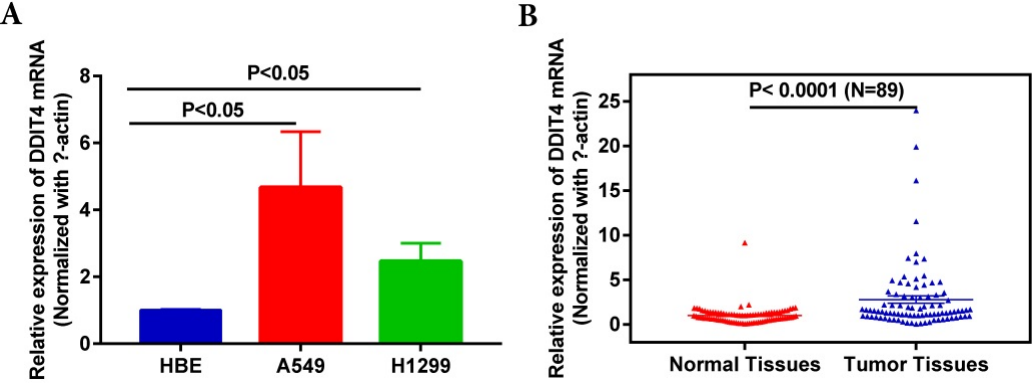
** Expression of DDIT4 is upregulated in LUAD cell lines and tissues. A.** DDIT4 expression is upregulated in LUAD cell lines. **B.** DDIT4 expression is upregulated in LUAD tissues.

To examine expression of DDIT4, we detected DDIT4 mRNA levels in two LUAD lines (A549 and H1299) and a human bronchial epithelial cell line (HBE). DDIT4 levels were significantly increased at the mRNA level in NSCLC cells compared to HBE cells (Fig. 1A). Then, we investigated the transcript levels of DDIT4 in 89 paired tumor and adjacent noncancer control tissues from LUAD patients by RT-PCR. The characteristics of the patients with LUAD were showed in Supplemental Table 1. The results showed that the mRNA levels of DDIT4 were significantly increased in tumor tissues compared to normal tissues (Fig. 1B).

### Immunohistochemical analysis of DDIT4 expression in lung LUAD

**Figure 2:**
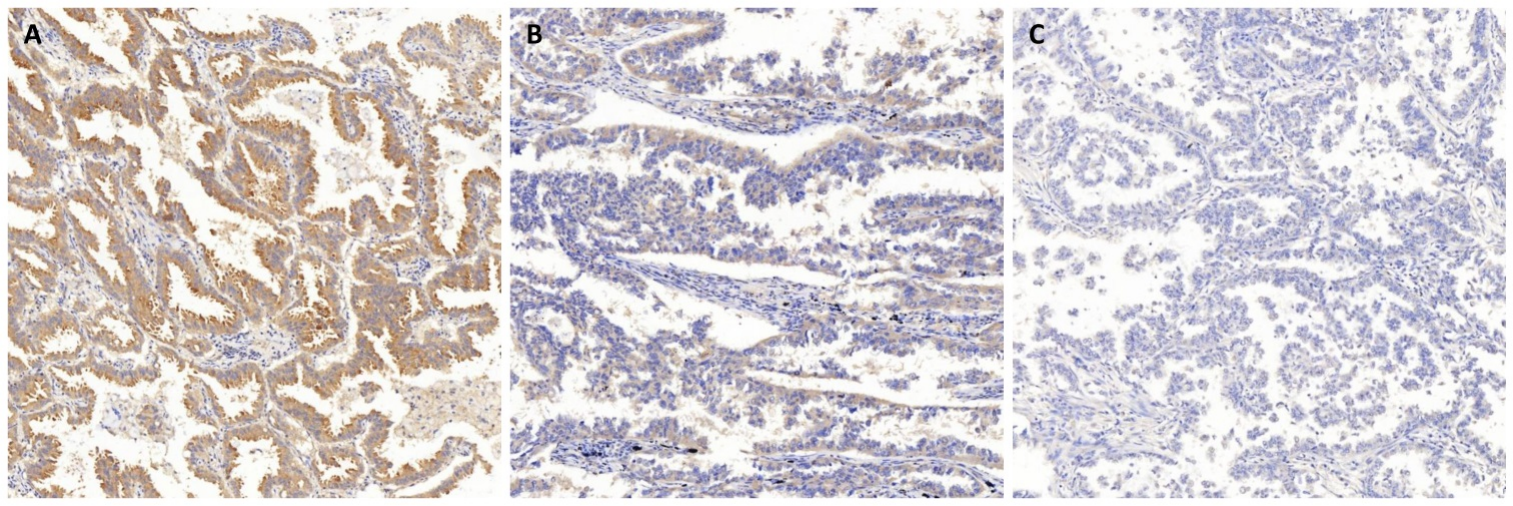
**Representative images of immunohistochemical staining of DDIT4 in lung adenocarcinoma tissue. A.** Strong positive staining for DDIT4 in LUAD. **B.** Medium positive staining for DDIT4 in LUAD. **C.** Negative staining for DDIT4 in LUAD (A, B, C: 100× magnification).

To investigate the associations between DDIT4 protein expression and clinical outcomes, we examined specimens from 75 cases of lung adenocarcinoma by immunohistochemistry (IHC) using anti-DDIT4 staining. Expression of DDIT4 was significantly increased in LUAD patients, whereas little or no DDIT4 expression was observed in normal lungs (Fig. 2A-C). These results suggest that there is a close relationship between DDIT4 and LUAD in which DDIT4 may participate in the pathological process of human LUAD. Intriguingly, DDIT4 expression was significantly correlated with differentiation (P < 0.05, Table 1). However, it was not correlated with sex, age, smoking, tumor size, lymph node metastasis, or TNM stage (P>0.05, Table 1).

### High DDIT4 expression correlates with shorter overall survival

**Figure 3:**
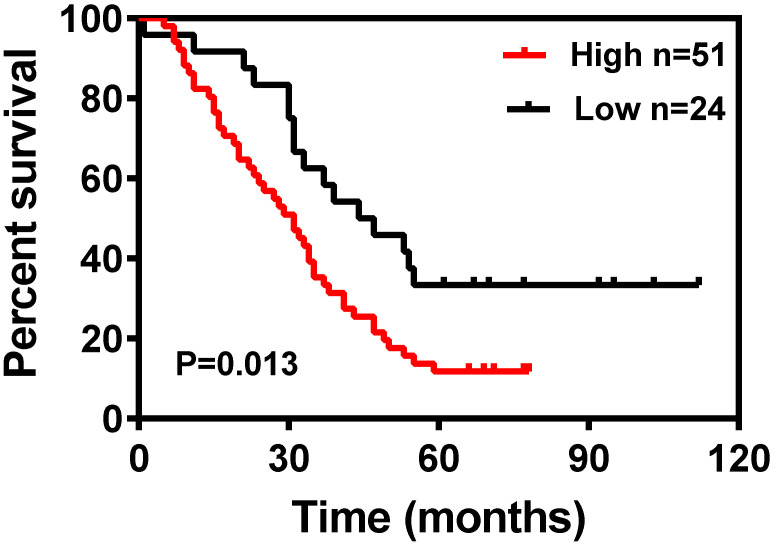
Kaplan-Meier survival curves for 75 LUAD patients according to DDIT4 expression status (log-rank test, P = 0.013).

The Kaplan-Meier survival curves for patients in the different categories of DDIT4 protein expression are shown in Fig. 3. High tumor expression of DDIT4 was significantly correlated with poorer overall survival in patients with LUAD (P < 0.05). In univariate analysis, sex, age, tumor size, lymph node metastasis, and TNM stage were not significantly associated with survival. Smoking (P < 0.001), differentiation (P < 0.001), and DDIT4 (P < 0.001) showed a statistically significant impact on survival (Table 2). Multivariate analysis revealed that smoking, differentiation, and DDIT4 were independent predictors of survival (Table 2).

### Evaluation of DDIT4 as a prognostic biomarker for NSCLC from the TCGA database

To evaluate the correlation between the mRNA level of DDIT4 and prognosis, a total of 70 LUAD patients with DDIT4 gene expression were followed up, and Progression-free survival (PFS) data were collected. Result of Kaplan Meyer curve revealed that LUAD patients with high DDIT4 expression had a significantly short PFS than those with low DDIT4 expression (P<0.01), and the result was showed in Supplemental Fig. S1. However, the relationship between overall survival (OS) and DDIT4 gene expression was not analyzed, because the follow-up time of most patients was less than 5 years and OS data cannot be completely collected.

To further verify the feasibility of the DDIT4 gene as a prognostic marker of lung cancer, the NSCLC dataset from TCGA was analyzed. Furthermore, we analyzed the correlation between DDIT4 expression and LUAD and DDIT4 expression and LUSC. RNA-seq gene expression data of 1025 samples (524 LUAD samples, 501 LUSC samples, and 108 normal tissue samples) generated by Illumina HiSeq were obtained from The Cancer Genome Atlas (TCGA). We set the median value as the dividing point for high expression and low expression of DDIT4 in the clinical analyses.

**Figure 4:**
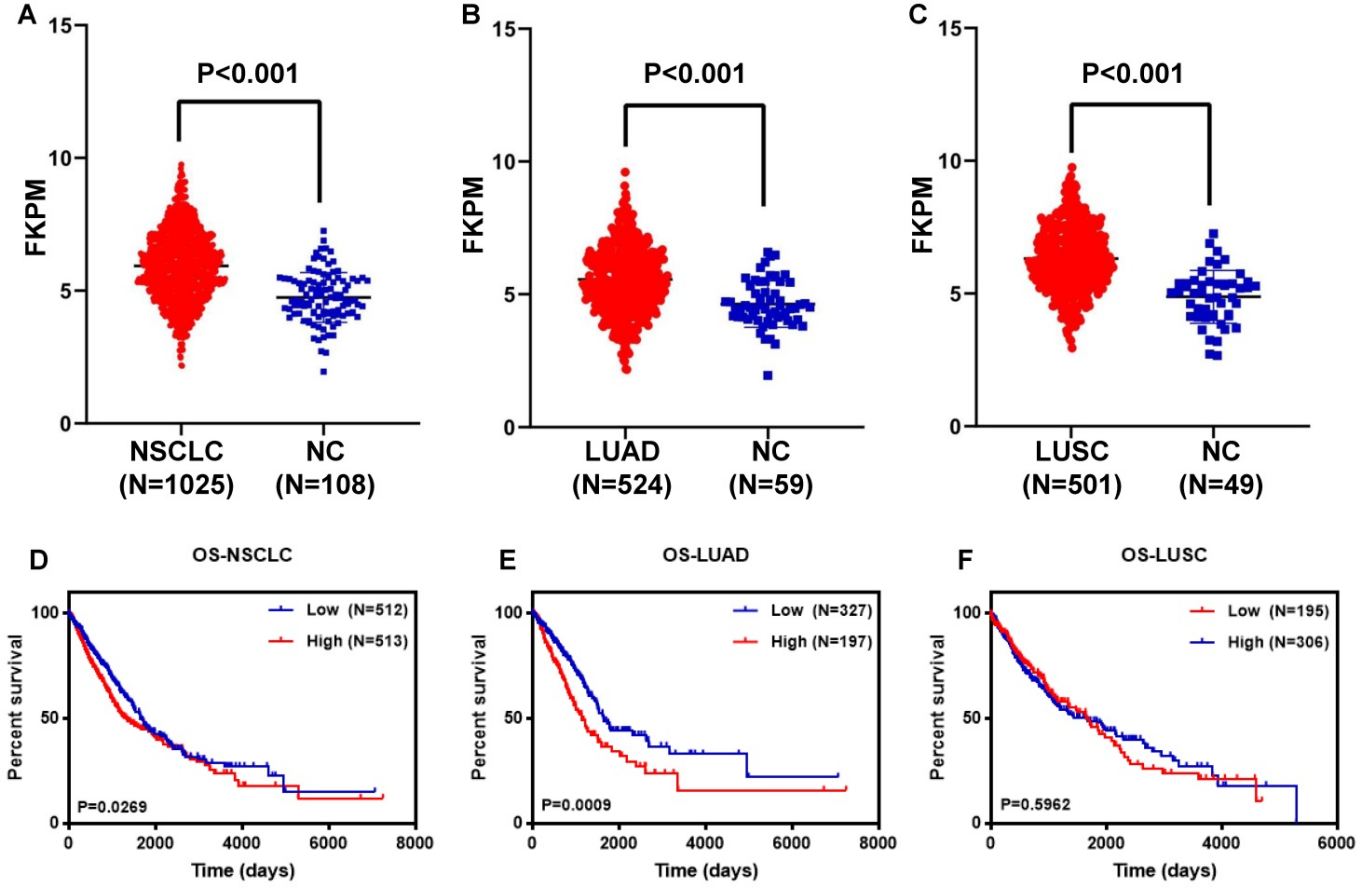
** Analysis of the DDIT4 gene in the TCGA database. A.** Expression of DDIT4 in NSCLC in the TCGA database. **B.** Expression of DDIT4 in LUAD in the TCGA database. **C.** Expression of DDIT4 in LUSC in the TCGA database. **D.** The relationship between the expression levels of DDIT4 and OS in NSCLC. **E.** The relationship between the expression levels of DDIT4 and OS in LUAD. **F.** The relationship between the expression levels of DDIT4 and OS in LUSC.

Statistical analysis revealed that DDIT4 was highly expressed in both lung adenocarcinoma and squamous cell carcinoma compared to noncancerous lung tissues (P < 0.001, Fig. 4A-C).

Further analysis revealed that LUAD patients with high DDIT4 expression had a significantly lower survival rate than those with low DDIT4 expression (P=0.0049, Fig. 4E).

### DDIT4 expression is upregulated in lung adenocarcinoma cell lines under hypoxia

**Figure 5:**
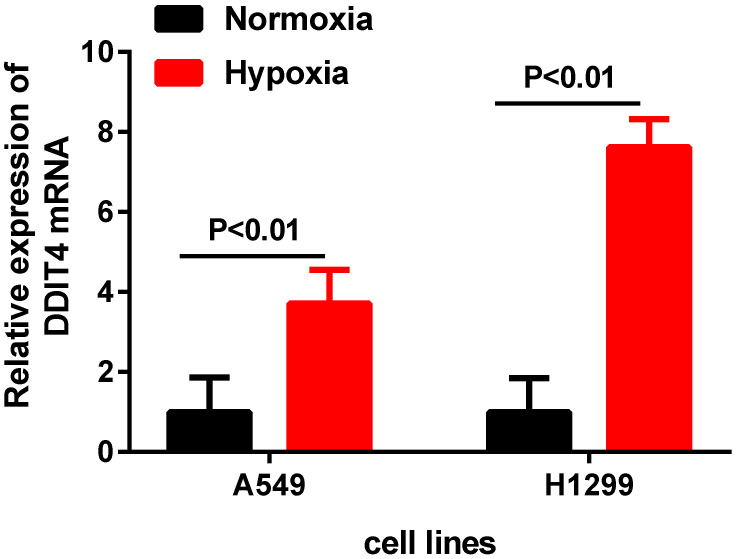
DDIT4 expression is unregulated in LUAD under hypoxia.

To further investigate the DDIT4 mRNA expression changes in LUAD cell lines under hypoxic conditions, we cultured two LUAD cell lines (A549 and H1299) under hypoxic conditions for 24 hours. The results showed that the mRNA levels of DDIT4 were highly increased under hypoxia compared to normoxia (P<0.01) (Fig. 5).

## Discussion

Lung cancer is one of the most common malignancies in the world 15. Due to tumor invasion and metastatic disease progression being more hidden in early stages, most patients with NSCLC are diagnosed in advanced stages. Therefore, it is of great significance to explore the mechanism of occurrence and progression of lung cancer to identify sensitive and specific lung cancer molecular markers. DDIT4 is induced by cellular stress conditions and regulates mTOR activity 7, and its abnormal expression has been linked to multiple diseases, including malignant tumors 16-17.

In this retrospective study, to investigate the role of DDIT4 in LUAD, we assessed the expression of DDIT4 in the H1299 and A549 LUAD cell lines. The results showed that DDIT4 expression was significantly higher in tumor cell lines than in HBE cell lines (Fig. 1A). Similarly, DDIT4 expression was significantly higher in LUAD tissues than in normal tissues (Fig. 1B). Additionally, immunohistochemical staining results demonstrated that high expression of DDIT4 was present in approximately 68.0% of LUAD tissues (Fig. 2). These findings indicate that DDIT4 is abnormally highly expressed in LUAD, consistent with a previous report by Jianyou Su et al. 18.

Next, we analyzed the relationship between DDIT4 expression and cytoplasmic and clinicopathological variables, which revealed that this gene may play a catalytic role in the occurrence and progression of LUAD (Tables 1, 2). Additionally, Kaplan-Meier survival analysis showed that high DDIT4 expression is correlated with shorter OS in 75 LUAD patients, and high DDIT4 expression is an independent prognostic factor for LUAD as analyzed by multivariate Cox proportional hazards regression (Fig. 3). In addition, we further analyzed the correlation between the mRNA level of DDIT4 and prognosis was analyzed. Kaplan Meyer curve revealed that high DDIT4 gene expression had a significantly short PFS than those with low DDIT4 expression in 70 LUAD patients (Fig. S1). However, the relationship between OS and DDIT4 gene expression was not analyzed, because the follow-up time of most patients was less than 5 years and OS data cannot be completely collected. We further searched the TCGA database and found that DDIT4 is correlated with the prognosis of lung cancer, especially in LUAD patients (Fig. 4). Patients with high DDIT4 expression levels exhibited poor prognosis (Fig. 3, Fig. S1, Fig. 4). Therefore, DDIT4 might be a prognostic biomarker for LUAD patients.

Schwarzer et al. found that expression of DDIT4 was significantly increased in hypoxic conditions, and the underlying mechanisms of REDD1 expression were dependent on PI3- kinase and could be further induced by hypoxia in a HIF-1a-dependent manner 19. In the present study, our RT-PCR results showed that hypoxia significantly enhanced the mRNA levels of DDIT4 in different LUAD cells compared to cells under normal oxygen conditions (Fig. 5), revealed that there is a certain relationship between DDIT4 and hypoxia stress response. However, the regulatory mechanism between hypoxia and DDIT4 remain to be further elucidated. Pascal Pineau et al. 20 identified DNA damage-inducible transcript 4 (DDIT4) as a direct target of miR-221 in hepatocellular carcinoma. Yan Li et al. 21 found that lysine-specific demethylase1 inhibitor named ZY0511 increased DDIT4 expression by altering H3K4mel 1/2 level of its promoter. In the future, abnormal expression and its regulation mechanism of DDIT4 gene in LUAD require further clarified. According to the previous study, DDIT4 also participated in the occurrence and development of tumors through other important pathways. Chang et al. 22 found that DDIT4 was a key mediator that participates in the RAS signaling pathway. Feng et al. 23 found that REDD1 expression and microvessel density count were positively correlated in oral squamous cell carcinoma, suggesting that REDD1 induction may stimulate angiogenesis. In fact, the regulatory mechanisms of DDIT4 involve complex crosstalk among different cellular signaling pathways 24. The biological roles of DDIT4 in LUAD need to further investigated.

In summary, our study demonstrated that DDIT4 is an independent predictor of overall survival in LUAD and has potential as a prognostic biomarker for non-small cell lung cancer in the future.

## Supplementary Material

Supplementary figure and table.

## Figures and Tables

**Table 1:** Relationship between expression levels of DDIT4 and clinicopathological features of 75 LUAD specimens

Variables	N	DDIT4		χ^2^	P
		Low	High		
**Sex**					
Female	35	10	25	0.355	0.552
Male	40	14	26		
**Age (years)**					
<65	44	12	32	1.093	0.296
≥65	31	12	19		
**Smoking**					
No	53	17	36	0.000	0.983
Yes	22	7	15		
**Lymph node metastasis**					
N0	35	14	21	1.93	0.165
N1/N2/N3	40	1	30		
**Differentiation**					
Well/moderate differentiation	40	17	23	4.43	0.037*
Poor differentiation	35	7	28		
**Tumor size (cm)**					
≤5	51	20	31	3.831	0.051
>5	24	4	20		
**TNM stage**					
I/II	46	18	28	2.780	0.095
III/IV	29	6	23		

Statistical analyses were performed using the Pearson χ^2^ test. P-values<0.05 were considered significant.

**Table 2:** Univariate and multivariate analysis of prognostic factors in 75 LUAD patients

Variables	Univariate analysis		Multivariate analysis	
	HR (95% CI)	P	HR (95% CI)	P
**Sex**				
Female	1.00	0.467		
Male	0.828 (0.499-1.376)			
**Age (years)**	1.00	0.242		
<65	0.732 (0.435-1.234)			
≥65				
**Smoking**				
No	1.00	0.028*	1.00	0.010*
Yes	1.837 (1.070-3.155)		2.085 (1.193-3.644)	
**Lymph node metastasis**				
N0	1.00	0.214		
N1/N2/N3	1.384 (0.829-2.313)			
**Differentiation**				
Well/moderate differentiation	1.00	0.013*	1.00	0.017*
Poor differentiation	1.898 (1.142-3.155)		1.902 (1.121-3.229)	
**Tumor size (cm)**				
≤5	1.00	0.776		
>5	0.922 (0.525-1.619)			
**TNM stage**				
I/II	1.00	0.062		
III/IV	1.640 (0.976-2.757)			
**DDIT4**				
Low	1.00	0.016*	1.00	0.041^*^
High	2.021 (1.137-3.590)		1.853 (1.025-3.349)	

Statistical analyses were performed by Cox proportional hazards regression. P-values<0.05 were considered significant. CI, confidence interval.
