# Supplementary material for: DDIT4 overexpression associates with poor prognosis in lung adenocarcinoma
Source: J Cancer. 2021 Sep 3;12(21):6422–8. doi: 10.7150/jca.60118 (PMC8489140; doi:10.7150/jca.60118)
Supplement: Supplementary file 1 — Supplementary figure and table. [file jcav12p6422s1.pdf]

Supporting Information

DDIT4 overexpression associates with poor prognosis in lung adenocarcinoma

Li Song[a,b†], Zhiyao Chen[a,†], Menghua Zhang[a], Mingli Zhang[a], Xiaoyun Lu[c], Chunsun Li[c,*] and Liyan Miao[a,*]

[a] Department of Clinical Pharmacology, The First Affiliated Hospital of Soochow University, Suzhou, Jiangsu, China, 215006.

[b] Department of Oncology, Affiliated Tumor Hospital of Nantong University, Nantong 226361, Jiangsu, China

[c] Department of Pathology, Affiliated Tumor Hospital of Nantong University, Nantong 226361, Jiangsu, China

[†] These authors contributed equally to this work.

[*]**Corresponding authors**

Liyan Miao, Chunsun Li

No. 188, Shi Zi Street, Suzhou, 215006, PR China

Tel.: (+86) 512-6797-2699; Fax: (+86)512-6797-2699

E-mail address: miaolysuzhou@163.com, miaoliyan@suda.edu.cn

Result of Kaplan Meyer curve revealed that LUAD patients with high DDIT4 expression had a significantly short progression-free survival than those with low DDIT4 expression (P<0.01).

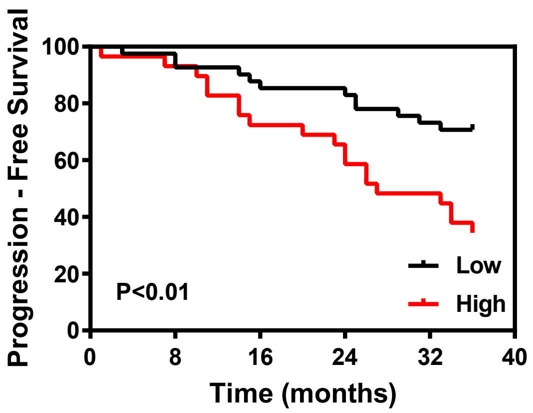

Fig. S1. The relationship between the expression levels of DDIT4 and PFS in LUAD (P<0.01)

Supplemental Table 1. Characteristics of patients with non-small cell lung cancer

	Lung adenocarcinoma (n=89)
Age, median (range)	68 (38-87)
Sex (n, %)	
Male	41 (46.1%)
Female	48 (53.9%)
Smoking status (n, %)	
Never smoker	66 (74.2%)
Ever smoker	23 (25.8%)
Lymph node metastasis	
N0	55 (61.8%)
N1/N2/N3	34 (38.2%)
Stage	
Stage I	34 (38.2%)
Stage II	24 (27.0%)
Stage III	27 (30.3%)
Stage IV	4 (4.5%)
